# Interaction of Polyanionic and Polycationic Brushes with Globular Proteins and Protein-like Nanocolloids

**DOI:** 10.3390/biomimetics8080597

**Published:** 2023-12-09

**Authors:** Tatiana O. Popova, Ekaterina B. Zhulina, Oleg V. Borisov

**Affiliations:** 1 Chemical Engineering Center, National Research University ITMO, 199004 St. Petersburg, Russia; salamatovat170301@gmail.com; 2 Institute of Macromolecular Compoundsof the Russian Academy of Sciences, 199004 St. Petersburg, Russia; kzhulina@hotmail.com; 3 CNRS, Université de Pau et des Pays de l’Adour UMR 5254, Institut des Sciences Analytiques et de Physico-Chimie Pour l’Environnement et les Matériaux, 64053 Pau, France

**Keywords:** protein absorption, polyelectrolyte brushes, Poisson-Boltzmann theory, electrostatic interactions, ionic strength

## Abstract

A large number of experimental studies have demonstrated that globular proteins can be absorbed from the solution by both polycationic and polyanionic brushes when the net charge of protein globules is of the same or of the opposite sign with respect to that of brush-forming polyelectrolyte chains. Here, we overview the results of experimental studies on interactions between globular proteins and polycationic or polyanionic brushes, and present a self-consistent field theoretical model that allows us to account for the asymmetry of interactions of protein-like nanocolloid particles comprising weak (pH-sensitive) cationic and anionic groups with a positively or negatively charged polyelectrolyte brush. The position-dependent insertion free energy and the net charge of the particle are calculated. The theoretical model predicts that if the numbers of cationic and anionic ionizable groups of the protein are approximately equal, then the interaction patterns for both cationic and anionic brushes at equal offset on the “wrong side” from the isoelectric point (IEP), i.e., when the particle and the brush charge are of the same sign, are similar. An essential asymmetry in interactions of particles with polycationic and polyanionic brushes is predicted when fractions of cationic and anionic groups differ significantly. That is, at a pH above IEP, the anionic brush better absorbs negatively charged particles with a larger fraction of ionizable cationic groups and vice versa.

## 1. Introduction

Over the past two decades, there has been significant interest in protein–polyelectrolyte complexes [1,2,3,4,5,6,7,8,9,10,11]. The studies on these systems have been motivated by multiple applications, including drug [12] and gene delivery, design of colloidal bionanoreactors [13,14], synthetic bio-adhesives [15,16,17], inhibition of viral infection, solubilization of components in the food industry [18,19], and protein purification [20,21,22], among others [23,24,25,26,27,28,29,30,31,32]. Additionally, it represents a cutting-edge approach for preventing non-specific protein adsorption [33] and facilitating protein immobilization [34].

Moreover, understanding the nature of protein–polyelectrolyte interactions is crucial for gaining insights into various biological processes. One notable example is the formation of natural chromosomes (DNA–histone complexes) [35,36]. In many cases, proteins interact with extracellular structures of strongly charged natural polyelectrolytes, e.g., glycosaminoglycans (GAGs). A typical example is aggrecan in articular cartilage, which can be envisioned as a polymer brush with keratan sulfate and chondroitin sulfate side chains end-tethered to the backbone formed by the core protein [37]. The interactions between these biopolyelectrolytes and proteins play a vital role in numerous biological processes [38,39,40,41,42].

In recent years, there has been a significant surge of interest in the field of biotechnology and medicine regarding the immobilization of proteins on solid supports. However, when biomolecules (e.g., enzymes and antibodies) adhere to solid surfaces, their biological function may be compromised or completely denatured [43,44]. At the same time, it was found that when biomolecules interact with polyelectrolyte brushes (that is, layers of charged macromolecules end-attached to planar substrates or surfaces of colloidal particles, immersed in aqueous solution), their enzymatic activity is usually preserved [1,45,46,47,48], and a large surface area is available for binding the appropriate number of molecules. Experimental studies have demonstrated that the enzymatic activity of, e.g., glucoamylase remains intact when adsorbed onto both strong and pH-sensitive polyelectrolyte brushes [13,14].

The polyelectrolyte brushes have also demonstrated a stabilizing effect, resulting in increased aggregative stability and the prevention of protein aggregation. Consequently, polyelectrolyte brushes represent a distinct class of colloidal nanostructures suitable for the effective immobilization of proteins [8,13,14,49]. The experimental and theoretical exploration of their interactions with charged species has been actively pursued (see ref. [38] for a comprehensive review). It has been convincingly demonstrated that, in spite of multiple types of interactions involved, the interactions between charged biomacromolecules and polyelectrolyte brushes are primarily governed by Coulomb forces.

In the realm of theoretical and experimental studies of polyelectrolyte (PE) brushes [50,51,52,53,54,55], it has been revealed that this layer predominantly accommodates a vast majority of its small mobile counterions. As a result, the brush layer itself adopts a state of near-neutrality, devoid of any significant excess charge. The presence of counterions within the brush layer not only contributes to its overall neutrality but also influences its height. The osmotic pressure, arising due to confinement of mobile counterions inside the PE brush, becomes the driving force for the swelling of the brush layer.

The driving force for protein adsorption on polyelectrolytes is often attributed to the release of counterions [56,57,58]. In this framework, the areas of positive/negative charge on the surface of proteins can be envisioned as multivalent counterions for polyelectrolyte (polyanionic/polycationic) chains [4,6,9,21,49,59,60,61,62,63,64,65]. As a result, the concomitant amount of small co- and counterions is released, and the entropy [9,47,66] of the entire system increases. This effect may counterbalance the repulsive Coulomb interactions as well as the steric repulsions between the proteins and the brush. It is important to note that, apart from the electrostatic attractions due to uneven (patchy) charge distribution on the protein surface, polyelectrolyte–protein binding can also take place on the “wrong” side of the protein isoelectric point (IEP) due to its re-ionization [47,59,67,68,69,70,71,72,73,74,75,76] in the strong local electrostatic field created by the polyelectrolyte chain or, particularly, by the PE brush. Hence, two mechanisms can drive the binding of proteins on the “wrong” side of IEP (i.e., by similarly charged polyelectrolytes). It is important to note that both the protein re-ionization (“charge regulation”) and patchy charge distribution on the surface of the protein globule may together contribute to the electrostatically driven protein uptake by the PE brush on the “wrong” side of protein IEP.

The ionic strength of the solution strongly affects the magnitude of protein absorption by the PE brush. The absorption of both similarly and oppositely charged, with respect to the brush, proteins is suppressed upon an increase in ionic strength [47,66]. At high ionic strength, the concentration of mobile ions is almost equal inside and outside the brush, and therefore the release of mobile counterions no longer leads to a significant increase in the system’s entropy. Furthermore, increasing the ionic strength decreases the Debye length, thereby reducing the range of electrostatic interactions. It is important to mention that aside from Coulomb interactions, other types of interactions, e.g., hydrophobic and steric ones, contribute to the overall protein–polyelectrolyte force balance. The exact mechanisms and factors influencing protein adsorption in this context are still an active area of research, and further studies are needed to fully understand the phenomenon.

## 2. Overview of Experimental Results

### 2.1. Interactions of Proteins with Strong or pH-Sensitive Anionic PE Brushes

The interaction of Bovine Hemoglobin (BHb) with strong polyelectrolyte spherical brushes of poly(styrene sulfonic acid) obtained through photoemulsion polymerization from a polystyrene core was studied in ref. [77]. The experiments were performed at pH = 7.2, which is slightly above the IEP (pI = 6.8–7.0). The location of hemoglobin molecules within the brush was determined using Small-Angle X-ray Scattering (SAXS), while Fourier-transform infrared spectroscopy (FTIR) was employed to analyze changes in the protein’s secondary structure inside the spherical brushes (SPBs). The results indicated that the proteins were able to penetrate deep into the brush, with approximately 30% of the proteins being absorbed on the surface of the polystyrene core due to hydrophobic interactions. The remaining proteins were found to be closely associated with the polyelectrolyte chains. Surprisingly, the authors concluded that there was no steric penalty for BHb penetration into the brush, despite the densely packed polyelectrolyte layer. Furthermore, it was demonstrated that the protein absorption process exhibits an entropic nature. Consistent with other studies (discussed below), a decrease in absorption was observed with an increase in salt concentration.

The findings presented in ref. [45] contrast with the previous observations. Here, the interaction between Bovine Serum Albumin (BSA) and Bovine Pancreatic Ribonuclease A (RNase A) with Spherical Brushes (SPBs) is observed. These SPBs were composed of a solid polystyrene core with a diameter of approximately 100 nm and featured long, densely grafted polyelectrolyte chains of either poly(styrene sulfonic acid, PSS) or poly(acrylic acid, PAA). The authors consider the “counterion release forces” to be the driving force of absorption on the “wrong side” of IEP.

In a series of experiments by Wittemann, Ballauff et al. [8,48,77], it has been demonstrated that protein (BSA) can undergo absorption into a weak polyelectrolyte brush layer, even when both the protein and the brush possess the same net charge (adsorption on the “wrong” side of the IEP), provided that the brush is in the osmotic limit, i.e., concentration of the counterions inside the brush exceeds salt concentration in the solution. In their subsequent studies, the same authors demonstrated that the amount of absorbed protein strongly depends on both the type of protein and the type of brush. They studied the absorption of several proteins, such as Bovine Serum Albumin (BSA), Bovine Pancreatic Ribonuclease A (RNase A), and β-lactoglobulin (BLG), on spherical weak (poly(acrylic acid)) and strong (poly(styrene sulfonate)) polyelectrolyte brushes. In the case of weak polyelectrolyte brushes, it was discovered that the ability to bind proteins was lower for all the studied proteins compared to strong polyelectrolyte brushes [48,49]. Additionally, the hydrophobic interaction between poly(styrene sulfonate) chains and hydrophobic regions of the protein should be considered. When comparing the degree of protein adsorption on the “wrong” side of the isoelectric point (IEP), it was observed that Bovine β-lactoglobulin (BLG) exhibits lower adsorption compared to Bovine Serum Albumin (BSA), while both proteins maintain their biological activity. Moreover, BLG demonstrates enhanced stability when absorbed by strong polyelectrolyte brushes. In subsequent work, it was also demonstrated that the structural integrity of proteins is maintained during and after immobilization on spherical quenched brushes [46].

The findings of W. Ouyang and M. Muller [78] present a contrasting perspective to the previously mentioned studies. Specifically, the authors of ref. [78] examined the interaction between proteins (such as Human Serum Albumin (HSA), Lysozyme (LYZ, hen egg), and Myoglobin (MYO, horse)) and spherical polyelectrolyte complex (PEC) particles.

These PEC particles consisted of a complex coacervate core formed by strong cationic poly(diallyldimethylammonium chloride) (PDADMAC) and either strong (namely poly(styrene sulfonate, PSS) or weak poly (maleic acid-co-α-methylstyrene, PMA-MS) polyanions. The polyanions were taken in excess and formed the solubilizing negatively charged corona of the PEC particles resembling a spherical polyelectrolyte brush (SPB). The PEC particles with a complex coacervate PSS/PDADMAC core and cationic PDADMAC corona were investigated as well. The authors observed that under conditions of electrostatic attraction that is below or above the protein IEP in the cases of negatively or positively charged PEC particles, flocculation occurred between the PEC particles and proteins. Consequently, the main focus was on studying the absorption of proteins on the “wrong” side of the isoelectric point (IEP) when both the protein and the PEC particle were similarly charged. Notably, the results highlighted that, in solutions with low ionic strength, particles with weak polyanionic coronas (PMA-MS) were more favorable for the absorption of proteins like LYZ, HSA, and MYO on the “wrong” side of the IEP compared to strong polyanionic (PSS) coronas. The observed phenomenon can be attributed to the higher molecular weight of PSS compared to PMA-MS and thicker negatively charged particle corona. It was also observed that when repulsion was present (similar charge of the protein and PEC particle), LYZ (Lysozyme) was absorbed less effectively on a polycation brush compared to HSA (Human Serum Albumin) and MYO (Myoglobin) on polyanionic brushes (PSS and PMA-MS). This difference could be attributed to the higher molecular weight and charge of the PDADMAC cationic corona, which resulted in stronger repulsion of the lysozyme. On the other hand, the lower mass of both polyanions resulted in a weaker repulsion of human serum albumin and myoglobin.

It has also been demonstrated in refs. [8,9,47] that, in addition to the type of the brush, the type of protein, pH of the solution, and ionic strength all have an impact on absorption. Specifically, the absorption of proteins on SPB is favorable at low ionic strength, while it is suppressed at a high ionic strength of the solution [79]. Hollmann and Czeslik [47] widely extend that study by comparing two different proteins, hen egg-white lysozyme and Bovine Serum Albumin (BSA). They demonstrated that both lysozymes, with a net positive charge, and BSA, with a net negative charge, can be absorbed onto PAA brushes attached to a planar substrate at low ionic strength. However, an increase in ionic strength facilitates protein release. In addition, the effect of pH on the degree of BSA adsorption was investigated. Consequently, as the pH of the solution increases (pH > 8), the absorption decreases due to the intense electrostatic repulsion between the BSA and the brush.

The influence of salt concentration on protein absorption in polyelectrolyte brushes has been substantiated through numerous experiments. Indeed, the adsorption is nearly completely reversible when the ionic strength of the solution is increased [9,10,55,80,81,82,83,84,85,86,87].

### 2.2. Interaction of Proteins with Anionic and Cationic Weak Polyelectrolyte Brushes

Wang et al. [88] explored the adsorption of BSA on weak polyanionic brushes and compared it to protein adsorption on cationic SPB. The researchers investigated the interaction of BSA with weak cationic poly (2-aminoethyl methacrylate hydrochloride) (PAEMH) and anionic poly(acrylic acid) (PAA) brushes under conditions of electrostatic attraction and repulsion, respectively. The findings of these studies revealed that both the cationic and anionic brushes exhibited absorption of BSA at pH = 6.1. However, the absorption was stronger in the case of the cationic brush, which can be attributed to an electrostatic attraction between the oppositely charged protein and brush. On the other hand, the absorption on the anionic brush was weaker, as both the protein in the solution and the brush carried the same charge sign. The study also highlighted that there is a saturation limit for the cationic brush in terms of BSA concentration, which is less than 10 g/L. The effect of salt on the adsorption process was evaluated. Similar to the anionic brush, an increase in ionic strength led to protein desorption from the cationic brush because of the screening of electrostatic interactions.

Wang and co-workers [89] also investigated the adsorption of β-lactoglobulin (BLG) and Bovine Serum Albumin (BSA) on spherical weak polycationic (PAEMH) and polyanionic (PAA) brushes using a combination of various techniques including turbidimetric titration, dynamic light scattering (DLS), zeta potential measurement, small angle X-ray scattering (SAXS), and isothermal titration calorimetry (ITC). It was observed that BLG showed better absorption on the “wrong” side of the isoelectric point (IEP) when using cationic brushes, whereas BSA exhibited better absorption with anionic brushes. This result was attributed to the difference in charge anisotropy on proteins with similar isoelectric points: BLG has a more pronounced negative spot, while BSA has a positive one, and this affects their interaction with the brushes. An investigation about the impact of salt on the absorption process revealed results similar to those obtained in previous studies.

### 2.3. Interaction of Proteins with Cationic Strong and Weak Polyelectrolyte Brushes

Zheng et al. [20] and Wang et al. [90] studied the selective absorption of different proteins using cationic strong and weak polyelectrolyte brushes, respectively. In ref. [20], the absorption of proteins like BSA, BLG, and HB on a strong cationic brush poly(methacryloxyethyltrimethyl ammonium chloride) (PDMC) was analyzed in terms of charge anisotropy. The results of this study demonstrate that a strong polycation brush can effectively separate proteins with distinct charge anisotropy, regardless of their isoelectric point, molecular weight, and structure. Specifically, the binding of BLG-SPB is significantly stronger than the binding of BSA-SPB, which was attributed to the visible negative charge spot on BLG. Furthermore, when comparing the binding of SPB-BSA and SPB-HB, it is evident that BSA is absorbed more efficiently due to the difference in isoelectric point (IEP). Altogether, these findings indicate that the affinity for protein binding on these SPBs follows the order: BLG > BSA > HB.

Wang et al. [90] studied the interaction between proteins such as Bovine Serum Albumin (BSA), β-lactoglobulin (BLG), and Papain, with weak spherical polycationic brushes of poly(2-aminoethyl methacrylate hydrochloride) (PAEMH). These cationic brushes demonstrated a stronger binding affinity for proteins in which the isoelectric point is <7 (BSA, BLG) compared to the protein, and for which IEP >7 (Papain). The absorption of these proteins onto the weak cationic brush followed the order: BLG > BSA > Papain. This can be attributed to the differences in protein size (molecular weight) and the distribution of charges on their surfaces. In other words, BLG has been absorbed more efficiently by the cationic brush due to its lower molecular weight compared to the other proteins (BSA, Papain). Additionally, BLG has pronounced negatively charged regions on its surface, unlike BSA and Papain. BLG with clear charge anisotropy started to be absorbed at a lower pH. Despite having almost identical isoelectric points (pI), BSA exhibited inferior absorption compared to BLG. This can be attributed to the less-pronounced charge anisotropy of BSA, which caused it to be absorbed by the polycationic brush at a pH higher than the pH at which BLG was absorbed. On the other hand, Papain bound weakly to the polycationic brush at a higher pH. It is worth noting that both BLG and BSA were absorbed on the “wrong” side of their isoelectric points, although the absorption was weak. Interestingly, BLG exhibited stronger absorption on the “wrong” side compared to BSA, which can be attributed to the earlier occurrence of a negative spot on the surface.

In ref. [91], a systematic study was conducted on the selective absorption of Bovine Serum Albumin (BSA) and β-glucosidase (β-G) by cationic weak (pH-sensitive) and strong spherical polyelectrolyte brushes (SPB). Two types of brushes were utilized, both consisting of a polystyrene core and a shell made of poly(2-aminoethylmethacrylate chloride) (PAEMH) and poly[2-(methacryloyloxy)ethyl] trimethylammonium chloride (PMAETA), respectively. The study revealed that the absorption of proteins by the brushes is influenced by the concentration of salt and the pH of the buffer. At low ionic strength, the absorption on strong polyelectrolyte brushes was found to be more efficient compared to that on pH-sensitive brushes. Furthermore, the protein-excluded volume effect was investigated using small-angle X-ray scattering (SAXS) analysis, which indicated that larger proteins (β-G) are likely to be absorbed in the outer layer of the brush.

Becker at al. [66] investigated the absorption of RNase A on strong spherical polycationic brushes derived from PMAETA. The technique employed for this analysis was isothermal titration calorimetry (ITC). The study revealed that, under low salt concentrations, RNase A tends to be absorbed on the “wrong” side of the isoelectric point (IEP) of the polycationic brushes. It is worth noting that all experiments related to the adsorption of RNase A on these brushes were carried out at a pH of 7.2, which is significantly below the enzyme’s isoelectric point. Furthermore, it was observed that even a slight increase in salt concentration impeded the absorption process, which is consistent with the predictions made by theoretical modeling [92]. The authors performed a van’t-Hoff analysis and discovered that the absorption is primarily governed by entropy. Data obtained from experiments performed at various salt concentrations indicated that the release of counterions contributes to the driving force behind the absorption of proteins on polyelectrolyte brushes with similar charges. Overall, this study provides valuable insights into the absorption behavior of RNase A on quenched spherical polycation brushes, shedding light on the roles of salt concentration and entropy in the process.

To summarize, extensive research has been conducted in the area of protein absorption on polyelectrolyte brushes, which has significantly contributed to the advancement of this field. However, to date, there have been no systematic comparative investigations on protein absorption on the opposite side of the isoelectric point (IEP) using cationic and anionic brushes with the same offset from the pI in the downward and upward directions, respectively.

## 3. Theoretical Model

Consider a protein-like nanocolloidal particle that comprises on its surface $N_{i+}$ ionogenic groups of type i+ capable of acquiring an elementary positive charge upon protonation, and $N_{j-}$ ionogenic groups of type j− capable of acquiring an elementary negative charge upon the dissociation of a hydrogen ion. The set of respective acidic ionization constants of cationic and anionic groups are $\left\{K_{i+},K_{j-}\right\}$, so that their degrees of ionization in the buffer solution with $pH_{b}\equiv -\log{\left[H^{+}\right]}_{b}$ are equal to

(1) $$\alpha_{bi+}={\left(1+K_{i+}/{\left[H^{+}\right]}_{b}\right)}^{-1}\equiv {\left(1+10^{pH_{b}-pK_{i+}}\right)}^{-1}$$

and

(2) $$\alpha_{bj-}={\left(1+{\left[H^{+}\right]}_{b}/K_{j-}\right)}^{-1}\equiv {\left(1+10^{pK_{j-}-pH_{b}}\right)}^{-1}$$

where $pK_{i+;j-}\equiv -\log K_{i+;j-}$.

The protein-like particle is interacting with a polyelectrolyte (PE) brush, which gives rise to excess electrostatic potential Ψ(z) at distance z≥0 from the grafting surface. The potential Ψ(z) is either a negative and monotonously increasing or positive and monotonously decreasing function of *z* in the cases of the anionic and cationic brush, respectively. The potential is calibrated as Ψ(z→∞)=0, that is, it vanishes in the solution far away from the brush. The explicit functional form of the potential Ψ(z) is presented in SI and depends on the architectural parameters of the brush (grafting density and polymerization degree of the brush-forming chains, as well as the fraction of charged monomer units, which is assumed to be quenched) and on the salt concentration $c_{s}$ in the solution. It is convenient to introduce the notation

(3) $$\lambda \left(z\right)\equiv \exp(e\Psi \left(z\right)/k_{B}T)$$

so that λ(z)≤1 and λ(z)≥1 for the polyanionic and polycationic brush, respectively.

Then, the degree of ionization of cationic and anionic groups of the nanoparticle in the potential Ψ(z) created by the brush is given by

(4) $$\alpha_{i+}\left(z\right)={\left(1+K_{i+}/\left[H^{+}\left(z\right)\right]\right)}^{-1}\equiv {\left(1+10^{pH\left(z\right)-pK_{i+}}\right)}^{-1}={\left(1+\frac{1-\alpha_{b+}}{\alpha_{b+}}\lambda \left(z\right)\right)}^{-1}$$

and

(5) $$\alpha_{j-}\left(z\right)={\left(1+\left[H^{+}\left(z\right)\right]/K_{j-}\right)}^{-1}\equiv {\left(1+10^{pK_{j-}-pH\left(z\right)}\right)}^{-1}={\left(1+\frac{1-\alpha_{b-}}{\alpha_{b-}}\lambda^{-1}\left(z\right)\right)}^{-1},$$

respectively. Here $\left[H^{+}\left(z\right)\right]$ is the local concentration of hydrogen ions at distance *z* from the grafting surface

(6) $$\left[H^{+}\left(z\right)\right]={\left[H^{+}\right]}_{b}\lambda^{-1}\left(z\right)$$

and ${\left[H^{+}\right]}_{b}$ is the concentration of the hydrogen ions in the buffer.

The free energy of the nanocolloidal particle in the electrostatic potential Ψ(z) created by the brush can be expressed as

$$F_{ion}\left(z\right)/k_{B}T=$$

$$\sum_{i+}N_{i+}\left\{\alpha_{i+}\left(z\right)\ln\alpha_{i+}\left(z\right)+\left(1-\alpha_{i+}\right)\ln\left(1-\alpha_{i+}\right)+\alpha_{i+}\ln\lambda \left(z\right)+\alpha_{i+}\ln K_{i+}-\alpha_{i+}\ln{\left[H^{+}\right]}_{b}\right\}+$$

(7) $$\sum_{j-}N_{j-}\left\{\alpha_{j-}\left(z\right)\ln\alpha_{j-}\left(z\right)+\left(1-\alpha_{j-}\right)\ln\left(1-\alpha_{j-}\right)-\alpha_{j-}\ln\lambda \left(z\right)-\alpha_{j-}\ln K_{j-}+\alpha_{j-}\ln{\left[H^{+}\right]}_{b}\right\}$$

Equation (7) comprises mixing entropies of ionized and non-ionized monomers (logarithmic terms), electrostatic free energies of positively and negatively charged groups, and their ionization free energies through respective ionization constants $K_{j-}$ and $K_{i+}=K_{w}/K_{base,i+}$ where $K_{w}=\left[H^{+}\right]\left[OH^{-}\right]=10^{-14}$; the last terms account for equilibrium with the reservoir with a fixed chemical potential of hydrogen ions $k_{B}T\ln{\left[H^{+}\right]}_{b}$ and hydroxyl ions $k_{B}T\ln{\left[OH^{-}\right]}_{b}=k_{B}T\left(\ln K_{w}-\ln{\left[H^{+}\right]}_{b}\right)$.

Minimization of the free energy, Equation (7), with respect to $\left\{\alpha_{i+},\alpha_{j-}\right\}$ leads to Equations (4) and (5) for position-dependent degrees of ionization of cationic and anionic groups. By substituting Equations (4) and (5) back into Equation (7), we obtain an expression for the free energy of the protein-like nanocolloid in the electrostatic field of the brush

(8) $$F_{ion}\left(z\right)/k_{B}T=\sum_{i+}N_{i+}\ln\left(1-\alpha_{i+}\left(z\right)\right)+\sum_{j-}N_{j-}\ln\left(1-\alpha_{j-}\left(z\right)\right)$$

The position-dependent differential free energy of transfer of the nanocolloid from the buffer solution into the brush is thus given by

$$\frac{\Delta F_{ion}\left(z\right)}{k_{B}T}\equiv \frac{F_{ion}\left(z\right)-F_{ion}\left(z\to \infty \right)}{k_{B}T}=$$

(9) $$\sum_{i+}N_{i+}\ln\frac{\left(1-\alpha_{i+}\left(z\right)\right)}{\left(1-\alpha_{bi+}\right)}+\sum_{j-}N_{j-}\ln\frac{\left(1-\alpha_{j-}\left(z\right)\right)}{\left(1-\alpha_{bj-}\right)}$$

The position-dependent net charge of the particle is given by

(10) $$Q\left(z\right)=\sum_{i+}N_{i+}\alpha_{i+}\left(z\right)-\sum_{j-}N_{j-}\alpha_{j-}\left(z\right)$$

The particle charge in the buffer

(11) $$Q_{b}=\sum_{i+}N_{i+}\alpha_{bi+}-\sum_{j-}N_{j-}\alpha_{bj-}$$

is negative or positive at $pH_{b}\geq pI$ or $pH_{b}\leq pI$, respectively, where pI is the isoelectric point at which the particle charge in the buffer vanishes, $Q_{b}\left(pH_{b}=pI\right)=0$.

As follows from the analysis of the position-dependent free energy, $\Delta F_{ion}\left(z\right)$, and particle charge, Q(z), the particle–brush interaction may follow three different scenarios depending on the sign and absolute value of the offset $\delta pH_{b}\equiv pH_{b}-pI$ from the IEP:

(i) The particle is attracted electrostatically by the polyanionic brush at $\delta pH_{b}\leq 0$ or by the polycationic brush at $\delta pH_{b}\geq 0$, that is, when the sign of the particle net charge (positive or negative, respectively) in the buffer, $Q_{b}$, is opposite to that of the brush. The insertion free energy $\Delta F_{ion}\left(z\right)$ is a negative and monotonously increasing function of *z*.

(ii) The particle is repelled electrostatically by the polyanionic brush at $\delta pH_{b}\gg 1$ or by a polycationic brush at $\delta pH_{b}<0$, $|\delta pH_{b}|\gg 1$, that is, when the particle net charge (negative or positive, respectively) in the buffer, $Q_{b}$, is sufficiently large by the absolute value and its sign is the same as that of the brush, the insertion free energy $\Delta F_{ion}\left(z\right)$ is a positive and monotonously decreasing function of *z*.

(iii) A delicate balance of electrostatic energy and free energy gain due to the re-ionization of the cationic and anionic groups of the nanoparticle leads to complex patterns in the insertion free energy $\Delta F_{ion}\left(z\right)$ at $\delta pH_{b}\geq 0$ or $\delta pH_{b}\leq 0$ and $|\delta pH_{b}|\leq 1$ for the polyanionic and polycationic brush, respectively. In this range of $\delta pH_{b}$, the free energy $\Delta F_{ion}\left(z\right)$ is an increasing function of *z* in the range of $0\leq z\leq z^{*}$, passes through a maximum at $z=z^{*}$, monotonously decreases at $z\geq z^{*}$, and then asymptotically approaches zero at z→∞. At the grafting surface, z=0, the free energy $\Delta F_{ion}\left(z=0\right)$ acquires either a negative or a positive value, which corresponds to a thermodynamically stable or metastable state of the particle localized inside the brush, respectively.

The particle net charge Q(z) is a monotonously increasing/decreasing function of *z* in the cases of the cationic/anionic brush, respectively, and it vanishes, changing its sign at $z=z^{*}$. Hence, the particle charged negatively/positively at $z\geq z^{*}$ becomes charged positively/negatively at $z\leq z^{*}$ upon insertion into the anionic/cationic brush. Therefore, the particle experiences repulsive/attractive force from the brush at $z\geq z^{*}$ and $z\leq z^{*}$, respectively.

The position $z^{*}$ of charge inversion (corresponding to the maximum in $\Delta F_{ion}\left(z\right)$) can be found from the condition $pH(z^{*})=pI$ that leads to

(12) $$\lambda \left(z^{*}\right)=10^{-\delta pH_{b}}$$

Hence, for given brush parameters and a given ionic strength of the solution, $c_{s}$, the position $z^{*}$ of the charge inversion point depends solely on the deviation $\delta pH_{b}$ (positive or negative in the cases of anionic/cationic brush, respectively) of the bulk $pH_{b}$ from the IEP. It is also worth noting that since the electrostatic potential profile protrudes outside the brush, the charge inversion may occur outside the brush if $|\delta pH_{b}|\ll 1$.

By substituting $\lambda (z^{*})$ given by Equation (12) into Equation (9) and with Equations (4) and (5), one finds the height of the potential barrier $\Delta F_{ion}\left(z^{*}\right)$ which appears to be independent of the brush parameters and of $c_{s}$ and is an increasing function of $|\delta pH_{b}|$ and depends on the set of $\left\{pH_{b}-pK_{i+,j-}\right\}$.

Since pI itself is a function of $\left\{N_{i+},N_{j-};pK_{i+},pK_{j-}\right\}$, the deviation $|\delta pH_{b}|$ is not a single parameter that controls the free energy patterns and the nanoparticle absorption/depletion scenario. Furthermore, for the same absolute value of deviation $|\delta pH_{b}|$ from the IEP, the free energy $\Delta F_{ion}\left(z\right)$ profiles for the same nanoparticle insertion into architectural, similar anionic ($\delta pH_{b}\geq 0$) and cationic ($\delta pH_{b}\leq 0$) brushes may differ.

Therefore, in order to unravel the relashipships of the protein-like nanoparticle interaction with polycationic and polyanionic brushes, we employ a simplified model of the particle with only one type of cationic and one type of anionic ionizable group, with respective numbers $\left\{N_{+},N_{-}\right\}$ and ionization constants $\left\{K_{+},K_{-}\right\}$.

If the fraction of cationic groups is defined as

$$f=\frac{N_{+}}{N_{+}+N_{-}}\equiv \frac{N_{+}}{N_{\Sigma }},$$

the isoelectric point can be found from the equation

(13) $$pI=pK_{-}+\log\left[\frac{2f-1}{2(1-f)}\frac{K_{-}}{K_{+}}+\sqrt{{\left(\frac{K_{-}}{2K_{+}}\right)}^{2}\cdot {\left(\frac{2f-1}{1-f}\right)}^{2}+\frac{f}{\left(1-f\right)}\cdot \frac{K_{-}}{K_{+}}}\right]$$

For the particular case f=0.5, Equation (13) is simplified as

(14) $$pI=(pK_{+}+pK_{-})/2$$

and

(15) $$\Delta F_{ion}\left(z\right)/\left[\left(N_{+}+N_{-}\right)k_{B}T\right]=\frac{1}{2}\ln\left(\frac{10^{-\Delta }+10^{\Delta }+10^{-\delta pH_{b}}+10^{\delta pH_{b}}}{10^{-\Delta }+10^{\Delta }+10^{-\delta pH_{b}}\cdot \lambda^{-1}\left(z\right)+10^{\delta pH_{b}}\cdot \lambda \left(z\right)}\right)$$

(16) $$Q\left(z\right)/\left(N_{+}+N_{-}\right)=\frac{1}{2}\left[\frac{10^{-\delta pH_{b}}\lambda^{-1}\left(z\right)-10^{\delta pH_{b}}\cdot \lambda \left(z\right)}{10^{\Delta }+10^{-\Delta }+10^{-\delta pH_{b}}\cdot \lambda^{-1}\left(z\right)+10^{\delta pH_{b}}\cdot \lambda \left(z\right)}\right]$$

where $\Delta =(pK_{+}-pK_{-})/2$, so that $pK_{+}=pI+\Delta$ and $pK_{-}=pI-\Delta$ and the potential barrier

(17) $$\Delta F_{ion}\left(z^{*}\right)/\left[\left(N_{+}+N_{-}\right)k_{B}T\right]=\frac{1}{2}\ln\left(\frac{10^{-\Delta }+10^{\Delta }+10^{-\delta pH_{b}}+10^{\delta pH_{b}}}{10^{-\Delta }+10^{\Delta }+2}\right)$$

**Figure 1:**
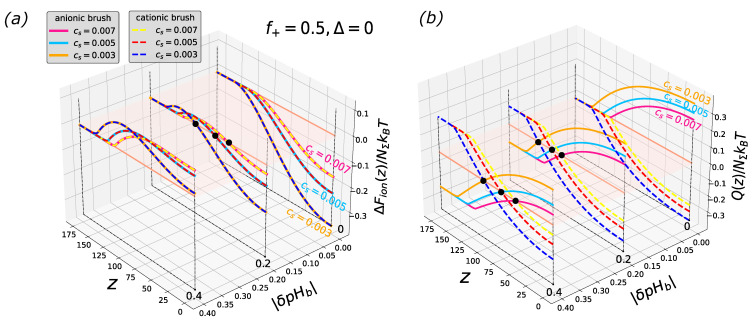
Cross-sections of the 2D profiles of the insertion free energy $\Delta F_{ion}(z,|\delta pH_{b}\left|\right)$ (**a**) and net charge $Q(z,|\delta pH_{b}|)$ (**b**) for polycationic/polyanionic brush at f=0.5; $\Delta =(pK_{+}-pK_{-})/2=0$ and three different salt concentrations $C_{s}=c_{s}a^{3}$, where $c_{s}$ is the number concentration of cations and anions of low molecular weight salt, and *a* is the monomer unit length for the brush-forming chains. The brush parameters are polymerization degree of the brush-forming chains N=300, reduced surface area per chain $S=s/a^{2}=100$, and fraction of permanently (positively or negatively) charged monomer units α=0.5. Black circles in panel (**a**) correspond to the coordinate of vanishing of the free energy, while black circles in panel (**b**) correspond to the charge inversion points. The red plane is a cross-section along the vertical axis at zero.

As follows from Equations (15) and (17), and as demonstrated in Figure 1, the free energy profiles of the particle insertion into a cationic and anionic brush for the same $|\delta pH_{b}|$ (i.e., with equal $pH_{b}$ offset above and below IEP in the cases of anionic and cationic brushes, respectively) exactly superimpose if the brushes are architecturally identical and differ only by the sign of their charge. This identity implies that ${\lambda \left(z\right)|}_{cationic}=\lambda^{-1}{\left(z\right)|}_{anionic}$. The profiles of the particle charge Q(z) are also identical with the accuracy of the sign.

Another important feature is that the height $\Delta F_{ion}\left(z^{*}\right)$ of the potential barrier to be overcome by the particle for entering a similarly charged brush (on the “wrong side” of the IEP) is independent of the configuration of the electrostatic field described by λ(z) and depends solely on deviation $|\delta pH_{b}|$ from the IEP and on the absolute value of $\left|\Delta |=|(pK_{+}-pK_{-})/2\right|$, being a decreasing function of |Δ|.

Therefore, as one can see in Figure 1, for a given $|\delta pH_{b}|\leq 1$, the depth of the free energy minimum $|\Delta F_{ion}\left(z=0\right)|$ at the grafting surface decreases upon an increase in salt concentration (leading to a decrease in |Ψ(z)|), whereas the free energy maximum becomes displaced towards the grafting surface but keeps a constant height $\Delta F_{ion}\left(z^{*}\right)$ irrespective of salt concentration.

As the salt concentration increases, the point at which the free energy vanishes and charge sign inversion occurs becomes shifted towards the grafting surface. These trends have been extensively examined in previous studies [92,93].

**Figure 2:**
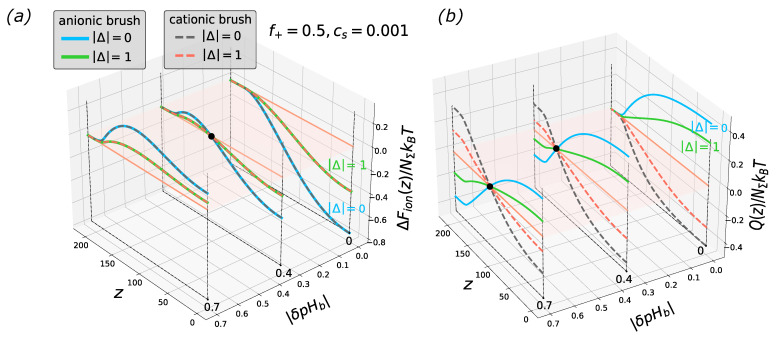
Cross-sections of the 2D profiles of the insertion free energy $\Delta F_{ion}(z,|\delta pH_{b}\left|\right)$ (**a**) and net charge $Q(z,|\delta pH_{b}|)$ (**b**) for polycationic/polyanionic brush at f=0.5 and salt concentration $C_{s}=0.001$ and two different values of $|\Delta =(pK_{+}-pK_{-})/2|=$ (0; 1) The brush parameters are as in Figure 1. Black circle in panel (**a**) corresponds to the coordinate of vanishing of the free energy, and black circles in the panel (**b**) correspond to the charge inversion points. The red plane is a cross-section along the vertical axis at zero.

Figure 2 illustrates that when the difference $\left|\Delta |=|(pK_{+}-pK_{-})/2\right|$ increases, the amplitudes of variations of both the free energy and net charge decrease, while the shape of the curves remains unchanged and they remain superimposed (with the accuracy of the particle charge sign) for cationic and anionic brushes irrespectively of the sign of Δ, that is, in the cases of $pK_{+}\geq pK_{-}$ and $pK_{+}\leq pK_{-}$.

When a particle’s surface contains unequal numbers of cationic and anionic groups, e.g., with a larger fraction of cationic groups, (f≥0.5), the free energy profiles for the polycationic and polyanionic brushes diverge. Additionally, the particle charge profiles in polycationic and polyanionic brushes are no longer symmetrical with respect to the *z*-axis.

**Figure 3:**
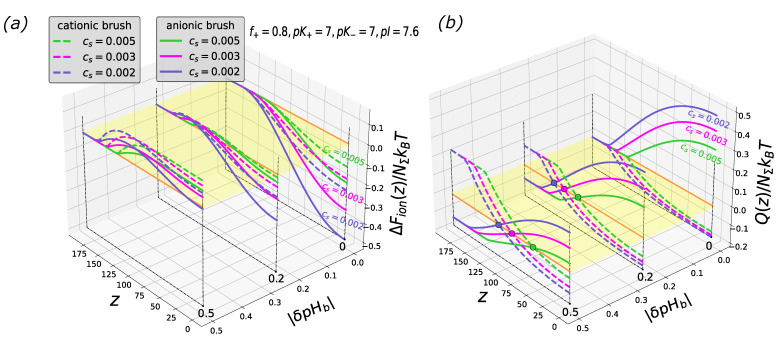
Cross-sections of the 2D profiles of the insertion free energy $\Delta F_{ion}(z,|\delta pH_{b}\left|\right)$ (**a**) and net charge $Q(z,|\delta pH_{b}|)$ (**b**) for polyanionic (solid lines) and polycationic (dashed lines) brush at f=0.8, and three different salt concentrations. $pK_{+}=pK_{-}=7$. The brush parameters are the same as in Figure 1. Colored circles in the panel (**b**) correspond to the charge inversion points. The yellow plane is a cross-section along the vertical axis at zero.

**Figure 4:**
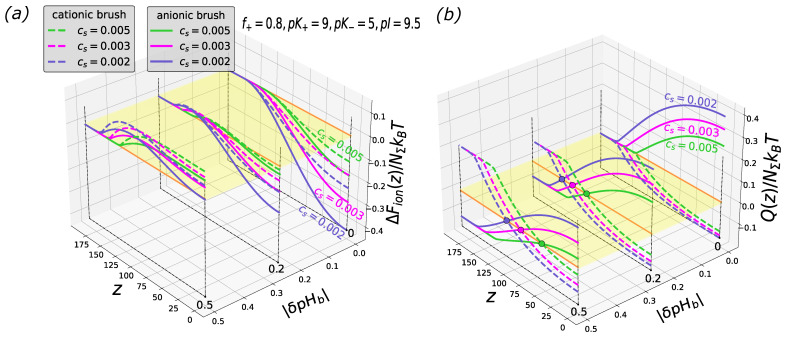
Cross-sections of the 2D profiles of the insertion free energy $\Delta F_{ion}(z,|\delta pH_{b}\left|\right)$ (**a**) and net charge $Q(z,|\delta pH_{b}|)$ (**b**) for polyanionic (solid lines) and polycationic (dashed lines) brush at f=0.8, and three different salt concentrations. $pK_{+}=9,\;pK_{-}=5$. The brush parameters are the same as in Figure 1. Colored circles in the panel (**b**) correspond to the charge inversion points. The yellow plane is a cross-section along the vertical axis at zero.

**Figure 5:**
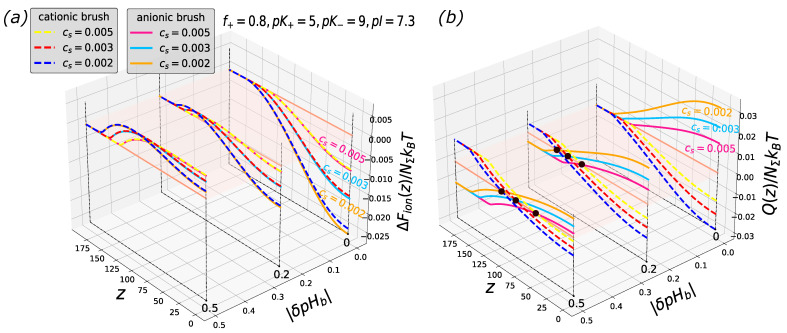
Cross-sections of the 2D profiles of the insertion free energy $\Delta F_{ion}(z,|\delta pH_{b}\left|\right)$ (**a**) and net charge $Q(z,|\delta pH_{b}|)$ (**b**) for polyanionic (solid lines) and polycationic (dashed lines) brush at f=0.8, and three different salt concentrations. $pK_{+}=5,\;pK_{-}=9$. The brush parameters are the same as in Figure 1. Black circles in the panel (**b**) correspond to the charge inversion points. The red plane is a cross-section along the vertical axis at zero.

In Figure 3, Figure 4 and Figure 5, the free energy and the net charge profiles of the particles with an asymmetric composition of cationic and anionic groups inserted into polyanionic and polycationic brushes are presented. As one can see from the Figure 3, Figure 4 and Figure 5, when cationic groups are present on the particles’ surface in a majority (f=0.8 is considered as a representative example), the polyanionic brush exhibits a better absorption capability compared to the polycationic brush both in the IEP and on the “wrong side” of it (at $pH_{b}$ above and below the IEP for polyanionic and polycationic brush, respectively).

This trend is manifested irrespectively of the values of the ionization constants of cationic, $K_{+}$, and anionic, $K_{-}$, groups.

However, the magnitude of variations in $\Delta F_{ion}\left(z\right)$ and Q(z) upon the insertion of the particle into the brush depends on the sign of $\Delta =(pK_{+}-pK_{-})/2$: At Δ≥0, the free energy and the particle charge profiles calculated for the same offset of $pH_{b}$ from the IEP, $pI(pK_{+},pK_{-},f)$, fairly superimpose with those for Δ=0. However, at Δ≤0, that is, when $pK_{+}<pK_{-}$, the magnitudes of variations in $\Delta F_{ion}\left(z\right)$ and Q(z) and, correspondingly, the depth of the potential well $|\Delta F_{ion}\left(z=0\right)|$ dramatically decrease, as seen in Figure 5. In the latter case, the particle is essentially uncharged as the $pH_{b}$ falls within a range below $pK_{-}$ and above $pK_{+}$.

## 4. Conclusions

Experimental data and theoretical considerations prove that a globular protein can be absorbed by both polyanionic and polycationic brushes in the vicinity of the IEP. At a pH above/below the IEP, the protein is readily taken up by a polycationic/polyanionic brush because of a Coulomb attraction to the oppositely charged brush, and the absorption is enhanced as the offset from the IEP increases. A more delicate balance of electrostatic forces and protein re-ionization in the electrostatic field created by the brush controls protein uptake on the “wrong side” of the IEP, that is, when the protein in the solution and the brush-forming chains carry charges of the same sign. Whether the absorption of any particular protein on the “wrong side” of the IEP is more efficient by a polyanionic or by a polycationic brush depends (apart of the difference in the brush electrostatic properties) on the particular composition of pH-sensitive cationic and anionic groups on the protein’s surface.

In order to examine this effect, we considered a simplified model of an ampholytic protein-like nanocolloidal particle interacting with either a cationic or anionic polyelectrolyte brush with identical architectures (i.e., the same polymerization degree of the brush-forming chains, grafting density, and fraction of permanently charged monomer units). Our self-consistent field analysis proves that the interaction patterns of protein-like nanoparticles comprising equal numbers of weak cationic and anionic ionizable groups with their respective ionization constants $K_{+},K_{-}$ and polyanionic/polycationic brush at pH>pI and pH<pI, respectively, are similar. The magnitude of attractive free energy minimum depends only on the absolute value of $\Delta =(pK_{+}-pK_{-})/2$ and decreases as |Δ| increases.

An asymmetry in the interaction between the polyanionic/polycationic brush and similarly charged protein-like nanoparticles emerges when the particle comprises essentially different numbers of cationic and anionic ionizable groups. For the same offset in pH from the IEP, a polyanionic/polycationic brush strongly absorbs the particle with the majority of cationic/anionic ionizable groups. Moreover, for the same value of |Δ|, the magnitudes of the free energy minima are notably larger at $pK_{+}\geq pK_{-}$ than at $pK_{+}\leq pK_{-}$, if cationic ionizable groups make up the majority. The opposite trend is expected when the majority of the ionizable groups on the particle surface are anionic.

We remark that there are other contributions to the insertion free energy, which do not depend on the signs of the particle and the brush charges, but depend on the absolute value of the electrostatic potential and profile of polymer density in the brush. They are (i) the osmotic repulsion proportional to the particle volume and arising due to an excess concentration of mobile counterions inside the brush; (ii) short-range non-electrostatic, e.g., hydrophobic, interactions between monomer units of the brush-forming chains and the particle [92]. These interactions may either hinder or enhance the particle uptake by the brush, but they provide the same contribution to the overall free energy of the nanoparticle/protein interaction for architecturally identical polycationic and polyanionic brushes. Finally, charge–charge correlations between monomer units of the brush-forming polyelectrolyte chains and oppositely charged “patches” on the protein globule surface may lead to additional asymmetry in the protein interactions with the polycationic/polyanionic brush. However, the latter effect has to be analyzed for each particular protein with its unique spatial structure. This is beyond the self-consistent field theory developed here, which is most accurate for proteins/nanocolloids with a fairly uniform surface distribution of cationic and anionic groups. In spite of simplifying the assumptions used, we believe that our theory captures important features in the asymmetry of interactions between protein-like nanoparticles and polycationic/polyanionic brushes and may serve as a guide for more systematic experimental research on well-defined model systems.
